# Current Status of Phytoseiid Mites as Biological Control Agents in Latin America and Experiences from Argentina Using *Neoseiulus californicus*

**DOI:** 10.1007/s13744-023-01026-4

**Published:** 2023-02-22

**Authors:** Carlos Vásquez, Yelitza Coromoto Colmenárez, Nancy Greco, Mayra Ramos

**Affiliations:** 1 Faculty of Agronomical Sciences, Technical University of Ambato, Campus Querochaca, Province of Tungurahua Cevallos, Ecuador; 2 CABI Latin America, Botucatu, São Paulo Brazil; 3 Centro de Estudios Parasitológicos y de Vectores (CEPAVE-CCT-La Plata-CONICET-UNLP), La Plata, Argentina; 4 Tecnológico Nacional de México, Campus Los Reyes, Colonia Libertad, Los Reyes, Michoacán México

**Keywords:** Phytoseiidae, Sustainable agriculture, Biocontrol agents

## Abstract

Phytoseiidae is a large family of Mesostigmata mites. Members of this family are important biological control agents across the world since they are well-known natural enemies of phytophagous arthropods on cultivated and non-cultivated plants, mainly used to control pest spider mites. However, some can control thrips in greenhouses and fields. Several studies reporting on species in Latin America have been published. The most extensive studies were conducted in Brazil. Phytoseiid mites have been used in different biological control approaches, with two successful classical biological control programs: the biocontrol of the cassava green mite using *Typhlodromalus aripo* (Deleon) in Africa and the citrus and avocado mites by *Euseius stipulatus* (Athias-Henriot) in California. Efforts in using phytoseiid mites to enforce biological control of different phytophagous mites are being made in Latin America. Till now, only a few successful examples are available on this topic. This fact highlights the need to continue the investigations on the ability of other unknown species to be used in biological control through close collaboration between researchers and biocontrol companies. Various challenges remain, such as developing better rearing systems to provide a large number of predators to farmers in various crop systems, training farmers to improve their understanding of the use of predators, and chemical control aimed at conservation biological control, looking forward to increasing the use of the phytoseiid mites as biological control agents in Latin America and the Caribbean.

## Introduction: an overview of the Phytoseiidae

Phytoseiidae is a large family of Mesostigmata mites, in which the number of species is being continuously revised. A total of 1500 species in 79 genera had been recognized in 1986, but these have increased to about 2300 species in 84 genera in 2007, while in 2012, 2692 species names were mentioned (including synonyms) (Demite et al. 2014, 2022).

Due to their predatory habits of feeding, Phytoseiid mites are important biological control agents all over the world since they are well-known natural enemies of phytophagous arthropods on cultivated and non-cultivated plants; mostly used to control pest spider mites, but some can control thrips and whiteflies in greenhouses and fields (Fathipour and Maleknia 2016). Although several other predatory mites are noted to control phytophagous mites, Phytoseiid mites have several advantages because of their high fecundity, abundant availability, good searching ability, dispersal rate, adaptability to different ecological niches, and a high degree of prey specificity (Gulati 2014). Besides, certain Phytoseiids consume large numbers of prey, maintain plant-feeding mites at low densities, and also have a rapid developmental rate comparable to their prey. Also, a female-biased sex ratio equivalent to their prey allows them to respond numerically to increased prey density, and can easily be mass-reared (Fathipour and Maleknia 2016).

Based on the diverse food sources used by Phytoseiid, McMurtry et al. (2013) proposed the new classification considering four lifestyles as follows: specialized mite predators (type I lifestyle), selective predators of tetranychid mites (type II lifestyle), generalist predators (type III lifestyle), and pollen feeding generalist predators (type IV lifestyle). Accordingly, Liu et al. (2017) showed a relationship between feeding habits and/or lifestyles of Phytoseiid based on variations in gnathostome morphology in which species are divided into three groups. Species in group II have larger chelicerae and hypostomes than those of groups I and III, while species in group III have larger lobes and angles of fixed digits than those of the other two groups. Although results were consistent with the previous classification, except that types I and II were not separated, authors indicated that feeding habits and lifestyles of Phytoseiids are also affected, apart from gnathostome morphology, by nutritional requirements, and digestive ability. It is necessary to evaluate the reliability of the method used by them and to discuss the possibilities for sub-grouping.

Considering the important role of Phytoseiid mites in biological control programs, they have received increasing attention worldwide. However, so far, there are as few as 30 species of predatory mites commercially produced as biological control agents (Liu et al. 2017; van Lenteren 2012). Also, *Phytoseiulus persimilis* (Athias-Henriot), *Neoseiulus cucumeris* (Oudemans), and *Amblyseius swirskii* (Athias-Henriot) have been successfully applied in many countries (Liu et al. 2017; van Lenteren 2012).

Based on the biological potential of Phytoseiid mites, and also due to the increasing requirements for alternatives to chemical control worldwide, more detailed studies should be addressed to investigate a large number of biological control candidates among this predatory mite family, mainly in Latin American countries where pest control is still based on chemical control.

## The biodiversity of Phytoseiid mites in Latin America

Mites of the family Phytoseiidae (Mesostigmata) have been extensively studied for their potential as biological control agents of phytophagous mites (McMurtry and Croft 1997).

Several studies reporting on the species in Latin America have been published; however, most of the works have been conducted in Brazil. According to Demite et al. (2022), Brazil has reported 259 species in 41 genera, with the higher number of species in the genera of *Amblyseius* (19.3%), *Neoseiulus* (10.4%), *Typhlodromips* (8.9%), *Phytoseius* (6.9%), and *Euseius* (6.6%), accounting 52.1% of the overall Phytoseiid diversity (Table 1).

**Table 1:** Total valid species of Phytoseiidae reported in Brazil, according to Demite et al. (2022)

Genus	Number of reported species
*Amazoniaseius*	1
*Amblydromalus*	13
*Amblyseiella*	1
*Amblyseius*	50
*Arrenoseius*	4
*Breviseius*	1
*Chelaseius*	3
*Cocoseius*	3
*Euseius*	17
*Galendromimus*	4
*Galendromus*	2
*Graminaseius*	3
*Honduriella*	1
*Ingaseius*	1
*Iphiseiodes*	12
*Iphiseius*	1
*Leonseius*	2
*Macrocaudus*	1
*Metaseiulus*	7
*Neoparaphytoseius*	3
*Neoseiulus*	27
*Paraamblyseius*	1
*Paraphytoseius*	3
*Phyllodromus*	1
*Phytoscutus*	2
*Phytoseiulus*	4
*Phytoseius*	18
*Proprioseiopsis*	12
*Proprioseius*	3
*Ragusaseius*	1
*Ricoseius*	1
*Scapulaseius*	1
*Serraseius*	1
*Silvaseius*	1
*Tenuisternum*	1
*Transeius*	6
*Typhlodromalus*	11
*Typhlodromina*	3
*Typhlodromips*	23
*Typhlodromus*	8
*Typhloseiopsis*	1

**Fig. 1:**
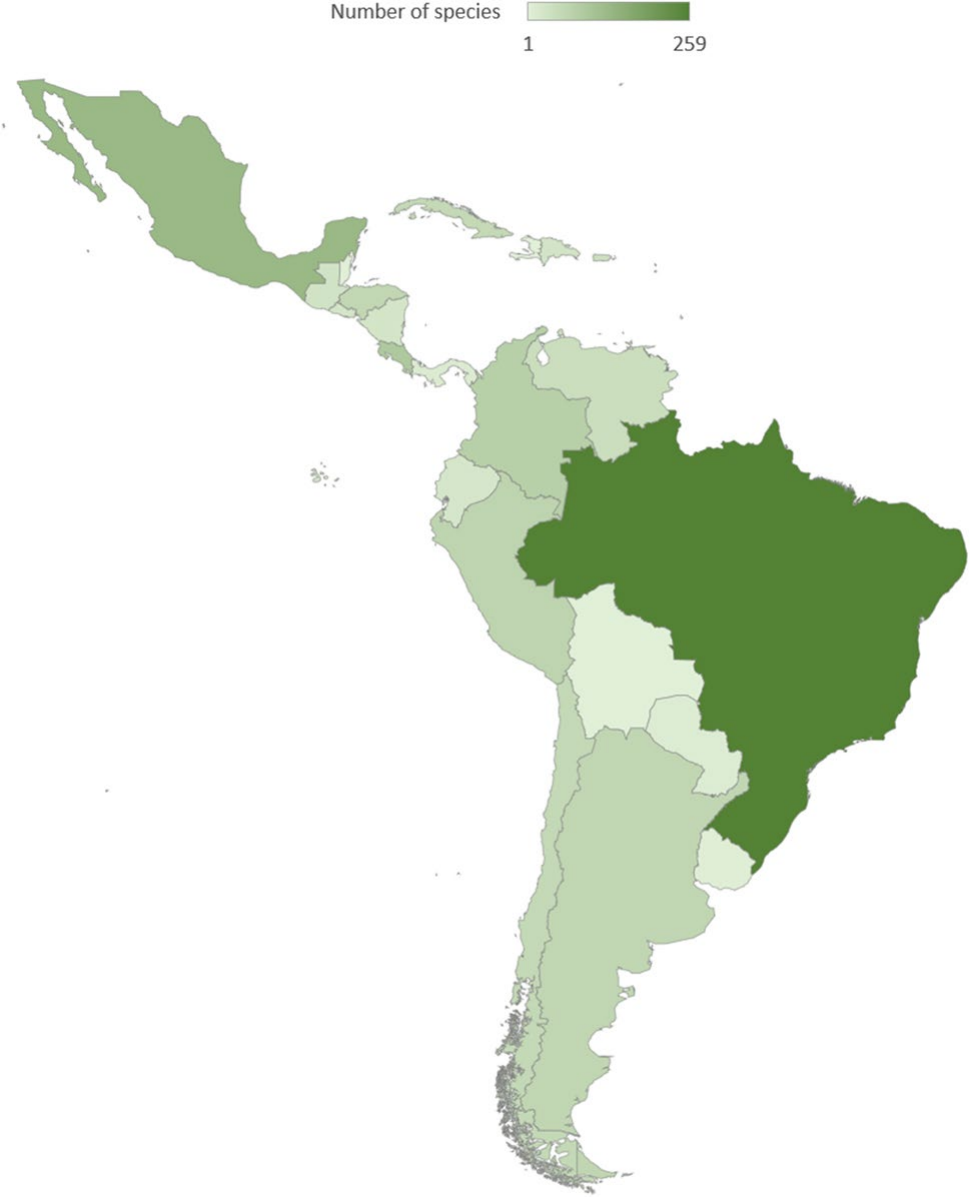
Number of Phytoseiidae species recorded in Latin American countries, according to Demite et al. (2022)

Conversely, a lower number of species have been reported in the different countries of Latin America (Fig. 1).

In Cuba, one of the first reports of Phytoseiidae mites was carried out by Smith and Summer (1949), who identified *Phytoseiulus macropilis* (Banks). Later, different studies have been done by Ramos and Rodríguez (2006), who provided the first list of this family in agroecosystems of Cuba, including new records (Table 2). de la Torre and Cuervo (2019) updated the list of Cuban mites, including 12 phytoseiid mites, but the crop or associated prey species were not stated (Table 2) by the author.

**Table 2:** The richness of Phytoseiidae species in agroecosystems of Cuba. From: Ramos and Rodríguez (2006) and de la Torre and Cuervo (2019)

Genus/species	Host plants	Associated to
Subfamily Amblyseiinae Muma		
* Amblydromalus limonicus* (Garman & McGregor)	–	–
* Amblydromalus manihoti* (Moraes)	–	–
* Amblydromalus manihoti* (Moraes)	–	–
* Amblyseius aerialis* (Muma)	*Citrus* spp., *Glycine max* (L.), *Cucumis sativus* Lin., *Solanum* t*uberosum* L	Tetranychidae
* Amblyseius aurences* (Athias-Henriot)	*Citrus* spp.	Tetranychidae
* Amblyseius curiosus* (Chant & Baker)	*Morus alba* L	*Thrips* spp.
* Amblyseius deleoni* (Muma)	*Citrus* spp.	Tetranychidae
* Amblyseius herbicolus* (Chant)	*Citrus* spp., *Solanum tuberosum* L	Not indicated
* Amblyseius largoensis* (Muma)	*Citrus* spp., *Solanum tuberosum* L	Tetranychidae Tarsonemidae Tenuipalpidae *Thrips* spp.
* Amblyseius lula* (Pritchard & Baker)	*Cocos nucifera* L	*Aceriaguerreronis* Keifer
* Amblyseius musae* (Garman)	*Musa* sp.	Not indicated
* Amblyseiusper longisetus* (Berlese)	–	–
* Amblyseius rhabdus* (Denmark)	*Fragaria* sp.	Tetranychidae, *Thrips* spp.
* Amblyseius solani* (Ramos & Rodríguez)	*Solanum tuberosum* L	Tarsonemidae
* Amblyseius sundi* (Pritchard & Baker)	*Citrus* spp., *Musa* sp.	Tetranychidae
* Amblyseius tamatavensis* (Bloomers)	*Musa* sp.	Tetranychidae
*Arrenoseius morgani* (Chant)		
* Euseius hibiscis* (Chant)	*Piper* sp., *Citrus* spp., *Mangifera indica* L., *Persea americana* Mill., *Psidium guajava L*	Tetranychidae
		Tarsonemidae
* Euseius vivax* (Chant y Baker)	*Carica papaya* L*.*, *Melicoccus bijugatus* Jacq	Tetranychidae
* Fundiseius hystric* (Muma)	*Solanum melongena* L	Tetranychidae
* Iphiseiodesquadripilis* (Banks)	*Citrus* spp.	Tetranychidae
* Iphiseiodeszuluagai* (Denmark & Muma)	*Achraszapota* L*.*, *Citrus spp*., *Psidiumguajava*L	Tetranychidae
		*Thrip*s spp.
*Neoseiulus alpinus* (Schweizer)		
* Neoseiulus anonymus* (Chant & Baker)	*Achras zapota* L*.*, *Citrus spp*., *Achyranthes aspera* L	Tetranychidae
* Neoseiulus anonymus* (Chant & Baker)	*Citrus* spp.	Tetranychidae
* Neoseiulus baraki* (Athias-Henriot)	*Oryza sativa* L	Tarsonemidae
* Neoseiulus californicus* (Mc Gregor)	*Saccharum spp.*	Tetranychidae
* Neoseiulus gracilis* (Muma)	*Cucumis sativus* L	Tetranychidae
*Neoseiulus lula* (Pritchard & Baker)		
Neoseiulus paraibensis (Moraes & McMurtry)	*Oryza sativa* L	Tarsonemidae
* Neoseiulus paspalivorus* (De León)	*Oryza sativa* L	Tarsonemidae
* Noelediusiphiformis* (Muma)	*Citrus* spp.	Tetranychidae
* Phytoscutussexpilis* (Muma)	Citrus spp.; *Solanum tuberosum* L	Tetranychidae
* Phytoseiulus macropilis* (Banks)	*Citrus* spp., *Musa* spp., *Fragaria* sp., *Ricinus communis* L., *Manihot esculenta* Crantz, *Phaseolus vulgaris* L., *Bidens pilosa* L	Tetranychidae
* Proprioseiopsis asetus* (Garman)	*Cucurbita pepo* L., *Chrysanthemum* spp., *Cucumis sativus* L., *Musa* spp.	Tetranychidae *Thrip*s spp.
* Proprioseiopsis cannaensis* (Muma)	*Chrysanthemum* spp.	Tetranychidae
* Proprioseiopsis elongatus* (Garman)	*Citrus* spp., *Mus*a spp., *Fragaria* sp., *Ricinus communis* L., *Manihot esculenta* Crantz	Tetranychidae
*Proprioseiopsis iphiformis*		
* Proprioseiopsis mexicanus* (Garman)	*Carica papaya* L	Tetranychidae
* Proprioseiopsis ovatus* (Garman)	*Citrus* spp.	Tetranychidae
* Proprioseiusmirandai* (De León)	*Manihot esculenta* Crantz, *Piper* sp.	Tetranychidae
* Typhlodromalus manihoti* (Moraes)	*Manihot esculenta* Crantz	*Thrip*s spp.
* Typhlodromalus peregrinus* (Muma)	*Citrus* spp*.*, *Oryza sativa* L	Tetranychidae
		Tarsonemidae
* Typhlodromips dentilis* (De Leon)	*Manihot esculenta* Crantz, *Solanum tuberosum* L*. Persea americana* Mill., *Bidens pilosa* L	Tetranychidae
Subfamily Phytoseiinae Berlese		
* Phytoseius ferox* (Pritchard & Baker)	–	–
* Phytoseius woodburyi* (De Leon)	*Piper* sp*.*, *Achyranthes aspera* L*.*, *Glycine max* (L.) Merr	*Thrip*s spp.
* Ricoseiusloxocheles* (De Leon)	*Citrus* spp.	Tetranychidae
Subfamily Typhlodrominae (Chant & McMurtry)		
* Africoseiulusnamibianus* (Ueckermann)	–	–
* Clavidromus transvaalensis* (Nesbitt)	*Citrus* spp*.*	Tetranychidae
* Galendromimus alveolaris* (De Leon)	*Citrus spp.*, *Hibiscus elatus L*	Tetranychidae
* Galendromus annectens* (De Leon)	*Citrus* spp.	Tetranychidae
* Galendromus floridanus* (Chant)	*Citrus* spp.	Tetranychidae
* Galendromus gratus* (Chant)	*Citrus* spp.	
* Galendromus longipilus* (Nesbitt)	*Citrus* spp.	Tetranychidae
* Metaseiulus ellipticus* (De Leon)	–	–
* Neoseiulella litoralis* (Swirski & Amitai)	–	*Thrip*s spp.
* Paraseiulellaeliptica* (De Leon)	*Citrus* spp.	Tetranychidae
* Phyllodromus leiodes* (De Leon)	*Waltheria americana L*	Eriophyidae, Tarsonemidae
* Typhlodromina conspicuus* (Garman)	*Citrus* spp.	Tetranychidae
* Typhlodrominaeharai* (Muma & Denmark)	*Citrus* spp.	Tetranychidae
* Typhlodromina subtropica* (Muma & Denmark)	*Citrus* spp.	Tetranychidae
* Typhlodromina tropicus* (Chant)	*Citrus* spp.	Tetranychidae
* Typhlodromus pilosus* (Chant)	–	–

Regarding other Latin American countries, in Venezuela, the most remarkable study was made by Aponte and McMurtry (1993) who reported 31 species of phytoseiid mites found in several areas of the country, of which 19 species had not been reported for Latin America (Table 3). Other contribution related to a characterization of the acarofauna associated with *Psidium guajava* reported to *Amblyseius tamatavensis*, *Euseius naindaimei*, *Proprioseiopsis ovatus*, *Neoseiulus caobae*, and *Typhlodromus paraevectus*, as the new records for *P. guajava* (Quirós et al. 2005).

**Table 3:** The main species of Phytoseiidae reported according to plant species in Colombia

Phytoseiid species	Host plant associated with phytoseiid mites
*Amblyseius anacardii*	*Citrus sinensis*
*Amblyseius aerialis*	*Clonorchis sinensis*, *Sida rhombifolia*, *Guazuma ulmifolia*, *Ipomoea batatas*, *Psidium guajaba*, *Banisteria* sp*.*, *Corchorus orinocensis*, *Vigna* sp., *Citrus* sp., *Morchella esculenta*
	*Passiflora edulis*
	*Gossypium hirsutum*
*Amblyseius arawak*	*Ficus benjamina*
*Amblyseius aciculus*	*Miconia* sp.
*Amblyseius chiapensis*	*Matisia cordata*
	*Citrus*
	sp. Trichantera
	*giant*, *Lagerstroemia* sp., *Bougainvillea glabra*, *Morchella esculenta*
*Amblyseius coffeae*	*Melicocca bijuga*
*Amblyseius curticerviscalis*	*Tricanthera* sp., *Plantago* sp.
*Amblyseius dentilis*	*Morchella esculenta*
*Amblyseius dominigos*	*Citrus* sp.
*Amblyseius farallonicus*	*Plantago* sp.
*Amblyseius fordycei*	*Citrus* sp.
*Amblyseius herbicolus*	*Citrus* sp*.*, *Inga spectabilis*, *Alsophila* sp., *Baccharis* sp., *Trichanthera gigantea*, *Miconia* sp., *Mangifera indica*, *Psidium guajava*, *Carpotroche pacifica*, *Saccharum officinarum*, *Coffea arabica*, *Morchella esculenta*
*Amblyseius largoensis*	*Clonorchis sinensis*
	*Psidium guajava*, *Morchella esculenta*
*Amblyseius deleoni*	*Clonorchis sinensis*
*Amblyseius gonzalezi*	*Morchella esculenta*
*Amblyseius lynnae*	*Andropogon* sp.
*Amblyseius manihoti*	*Morchella esculenta*, *Coffea* sp., *Bixa orellana*, *Citrus* sp., *Mikonia* sp.
*Amblyseius miconiae*	*Miconia* sp.
*Amblyseius neotropicus*	*Morchella esculenta*, *Psidium guajava*, *Ctirus* sp.
*Amblyseius neoperditus*	*Miconia* sp., *Caladium macrophytes*, *Citrus* sp., *M. esculenta*
*Amblyseius pentagonoalis*	*Calopogonio muconoides*, *Coffea* sp.
*Amblyseius sinenses*	*Psidium guajava*
*Amblyseius vasiformis*	*Citrus* sp., *Matisia cordata*, *Psidium guajava*, *Mangifera indica*, *Morchella esculenta*
*Cydnodromella alveolaris*	*Morchella esculenta*
*Diadromus regularis*	*Citrus lemon*

Similarly, the first studies in Colombia were made by Denmark and Muma (1972) who reported 11 species, describing two new species (*Iphiseiodes zuluagai* Denmark and Muma, on Citrus sp., and *Passiflora edulis* and *Typhlodromips sinensis* Denmark and Muma, on *Citrus sinensis* L.). In a second report of the phytoseiids of Colombia, Moraes et al. (1982) described a new genus and species*, Quadromalus colombiensis* and *Euseius ricinus* n. sp., bringing a total of 17 species to this country. Later, the number of species increased to 36, mainly belonging to *Amblyseius* (20 spp.), followed by *Euseius* (6 spp.), and *Typhlodromus* (5 spp.) (Moraes and Mesa 1988). Other less diverse genera included *Phytoseius* (2 spp.), *Cydnodromella* (1 sp.), *Paraphytoseius* (1 sp.), and *Typhloseiopsis* (1 sp.) with the description of three new species (*Amblyseius bellottii* from *Banisteria* sp., *Amblyseius caliensis* from *Miconia* sp., and *Typhloseiopsis neopritchardi* from *Capsicum frutescens*). The preponderance of *Amblyseius* species in Colombia was confirmed in a review made by Valencia et al. (2022) who contributed a list of main host plants to Phytoseiidae in Colombia (Table 3).

Besides, González Cano et al. (2016) carried out samplings to identify Phytoseiid species associated with fruit trees in Valle del Cauca, and a total of 16 species [*Amblyseius aerialis* (Muma), *Amblyseius chiapensis* De Leon, *Amblyseius herbicolus* (Chant), *Amblyseius tamatavensis* Blommers, *Euseius alatus* De Leon, *Euseius concordis* (Chant), *Euseius naindaimei* (Chant & Baker), *Euseius sibelius* (De Leon), *Galendromus* (*Galendromus*) *annectens* (De Leon), *Iphiseiodes zuluagai* Denmark & Muma, *Neoseiulus anonymous* (Chant & Baker), *Neoseiulus californicus* (McGregor), *Neoseiulus neotunus* (Denmark & Muma), *Phytoseius purseglovei* De Leon, *Typhlodromalus aripo* De Leon, *Typhlodromina tropica* (Chant)] associated to 17 fruit tree species including *Annona cherimola* Mill., *Annona muricata* L., *Annona reticulata* L., *Annona squamosa* L., *Borojo apatinoi* Cuatrec., *Carica papaya* L., *Citrus* sp., *Chrysophyllum cainito* L., *Fragaria* spp., *Mammea americana* L., *Mangifera indica* L., *Matisia cordata* Bonpl., *Passiflora edulis* Sims., *Prunus dulcis* L., *Psidium guajava* L., *Theobroma cacao*, and *Vitis vinifera* L. Based on the wide diversity of Phytoseiidae in this region, authors concluded that more studies are necessary to determine the potential of these natural enemies to be included in the biological control programs.

In other Caribbean islands, few are still known in most of the region. In the Dominican Republic, Ferragut et al. (2011) surveyed areas of natural vegetation and they reported 23 species, with *Phytoseius dominicensis* (Ferragut & Moraes) and *Typhloseiopsis adventitius* (Ferragut & Moraes) being described as the new taxa to science. In Puerto Rico, Denmark and Muma (1975) listed 48 species, including three new species named *Typhlodromips plumosus*, *Typhlodromalus higuilloae*, and *Amblydromella deleoni*. A more discrete number of species have been recorded in other Caribbean islands such as Belize (3 spp.), Antigua (2 spp.), Barbados, Bermuda, Haiti, and Saint Kitts with one species each (Demite et al. 2014).

## Biological control of Phytophagous mites in Latin America: a brief comparison to other successful cases around the world

Phytoseiid mites have been used in different biological control approaches. In classical biological control programs, there are two long-term and successful projects based on the use of these predatory mites: the cassava green mite in Africa and the citrus and avocado mites in California. In the case of the cassava green mite, *Mononychellus tanajoa*, several phytoseiid species were introduced and released in Africa; however, only *Amblydromalus manihoti* (Moraes) and *Typhlodromalus aripo* De Leon were set (McMurtry et al. 2015). According to these authors, *T. aripo* showed a higher potentiality for pest control due to the high dispersal and consumption rates, and ability to persist in cassava fields, provoking a reduction of the pest population, which produced direct economic benefits of US$1.7, 74, and 384 billion for Nigeria, Benin, and Ghana, respectively (McMurtry et al. 2015).

Experiences in biological control of tetranychid mites in citrus and avocado in California reported more than 25 re-introduced and released species; however, only *Euseius stipulatus* has spread extensively from release sites four decades after releases (McMurtry et al. 2015). Consequently, *E. stipulatus* was introduced to and currently produced commercially in Peru in 2006 for the control of the citrus red mite.

A comparative study to evaluate the efficiency of two *Amblyseius largoensis* populations (La Réunion and the state of Roraima, Brazil) in controlling the invasive tenuipalpid mite, *Raoiella indica*, was carried out in Brazil; however, none of the populations exhibited efficacy to control the pest (Morais et al. 2016). These findings suggested the necessity of complementary studies under conditions as close as possible to those of natural fields to imitate the natural environment for fully grown plants and to increase the predation rate. Although *A. largoensis* is a promissory biological control agent for combating *R. indica*, this predator has not yet been commercially available, hence, the potential of *Neoseiulus barkeri* Hughes, a predator commercially available in Brazil, was evaluated against *R. indica*. Thus, the higher consumption and reproduction rates of *N. barkeri* fed on *R. indica* suggested *N. barkeri* is an effective predator to control the red palm mite as part of a biological control program (Filgueiras et al. 2020a, b). Moreover, according to Filgueiras et al. (2020c), this predatory mite showed a preference for eggs over other stages of the prey, which fitted a type II functional response. Accordingly, the higher the prey density, the higher number of eggs laid by *N. barkeri* females, but it tended to stabilize when the number of *R. indica* was greater than 80, suggesting that *N. barkeri* shows to be promissory in control *R. indica* populations. However, further studies are needed.

Concerning conservation biological control, generalist predatory mite species naturally occurring in agroecosystems, both on crops and adjacent natural vegetation, can be used to manage phytophagous mite populations. Tixier (2018) took some examples from three species, *Kampimodromus aberrans*, *Euseius stipulatus*, and *Typhlodromus* (*Typhlodromus*) *pyri* in vine and citrus crops, and the main lesson learned is that the occurrence database can help in determining the probability of finding predatory mite species on crops and non-crop plants.

Laboratory experiments carried out in Rio Grande do Sul, Brazil, demonstrated that *Neoseiulus idaeus* (Denmark & Muma) completed its development when feeding on *Mononychellus planki* (McGregor), *Tetranychus ludeni* (Zacher), and *Tetranychus urticae* (Koch), suggesting that this predator mite may be utilized in biological control; however, further studies are required to define predator’s performance in the field (Reichert et al. 2017). Additionally, since *N. idaeus* is commonly found in semiarid regions of South America, laboratory assays compare the predation and oviposition rates of a drought-tolerant strain with the performance of a commercial species, *Neoseiulus californicus* (McGregor) (Sousa-Neto et al. 2021). Drought-tolerant *N. idaeus* showed a higher predation rate and similar oviposition performance as compared to *N. californicus*, and in addition, *N. idaeus* showed a higher searching rate even at low prey density on cowpea, suggesting that *N. idaeus* is a good candidate to control *T. urticae*, especially in semiarid regions.

Experiences in using phytoseiid mites to control pests other than mites have also been reported. Cavalcante et al. (2017) evaluated the potential of a Brazilian population of *Amblyseius tamatavensis* to manage *Bemicia tabaci* populations on artificially infested bell pepper (*Capsicum annuum* L) plants under laboratory conditions (28 ± 1 °C, 80 ± 10% RH, and 12 h of daily photoperiod). When released on artificially infested bell pepper plants, *A. tamatavensis* completed immature development in 5.1 days with an oviposition mean rate of 1.0 eggs/female/day, reducing the population density of *B. tabaci* by 60 to 80%, indicating that this phytoseiid mite is a promising biological control agent of *B. tabaci* biotype B.

Efforts in using phytoseiid mites to implement biological control of different phytophagous mites are being made in Latin America; however, more detailed laboratory and field studies are required to improve our knowledge about rearing and release techniques, habitat management strategies, susceptibility to agrochemicals, predation efficiency, interaction with other organisms of the agroecosystem, among others.

## Current status of predatory mites as biological control agents in Latin America

Predatory mites, mainly from the family Phytoseiidae (Acari: Mesostigmata), are used to control mite pests, as well as small insects throughout the world. The Phytoseiidae family contains more than 2692 species and the countries with the highest number of reported species are the USA, China, India, Brazil, and Pakistan (Demite et al. 2022). Some of these species are used as agents by all three widely recognized biological control strategies: (1) classical biological control to control, invasive pests introducing them in the targeted area, from the pest origin region (De Clercq et al. 2011); (2) augmentative biological control that consists of a mass release of the exotic or endemic, biological control agent in crops (van Lenteren 2012, 2017); and (3) conservation biological control that consists of enhancing the performance of natural enemies in the crops through the agroecosystem management (Tixier 2018).

Around 38 species of predatory mites are commercially produced in the world, representing 13% of all arthropods used (van Lenteren et al. 2017). The mites most used as the control agents are *Phytoseiulus persimilis* (Athias-Henriot) (type I lifestyle) and *Neoseiulus californicus* (McGregor) (type II lifestyle) for the control of the two-spotted spider mite *Tetranychus urticae* (Koch), and *Amblyseius swirskii* Athias-Henriot (type III lifestyle) for control of thrips and whiteflies in greenhouses. In Latin America, 10 species of Phytoseiidae have been used in different countries, from their region of origin or another, through classic biological control programs and, sometimes, in combination with augmentative biological control (Table 4). Several species (about 14), exotic and native, are used by this last strategy (van Lenteren et al. 2020). Most predatory mites are food-generalists, which can feed on many prey and plant exudates, pollen, and fungi, and they are naturally present both on crops and on adjacent natural vegetation. Such characteristics make them good candidates for conservation biological control implementation, so several studies have advanced in this sense, describing the relationship of Phytoseiid with the natural vegetation surrounding crops (Tixier 2018).

**Table 4:** Phytoseiidae used by classic biological control (CBC) and/or augmentative biological control (ABC) projects in Latin America

Country	Phytoseiid mite	Pest	Strategy	Source
Argentina	*Neoseiulus californicus* *Iphyseius degenerans*	*Tetranychus urticae* Trips	CBC CBC	Greco et al. (2020)
Brazil	*Amblyseius tamatavensis* *Euseius concordis* *Neoseiulus anonymus* *Neoseiulus californicus* *Neoseiulus barkeri* *Neoseiulus idaeus* *Phytoseiulus macropilis* *Phytoseiulus longipes*	*Tetranychus urticae*	ABC ABC ABC	Bueno et al. (2020), Cavalcante et al. (2017), Sousa-Neto et al. (2021), Massaro et al. (2016)
Chile	*Neoseiulus californicus* *Neoseiulus cucumeris* *Phytoseiulus persimilis*	*Panonychus ulmi*, *Panonychus citri*, *Polyphagotarsonemus latus*, *Oligonychus yothersi*, *Tetranychus urticae*, *Tetranychus cinnabarinus*, *Brevipalpus chilensis* Several species of “thrips” *Tetranychus urticae*	ABC ABC ABC	SAG, Chile (2022)
Colombia	*Neoseiulus californicus* *Neoseiulus cucumeris* *Neoseiulus barkeri* *Phytoseiulus persimilis* *Galendromus* (= *Thyplodromus*) *occidentalis*	*Tetranychus urticae* *Panonychus citri* *Panonychus ulmi* *Phytonemus pallidus* *Polyphagotarsonemus latus* *Raoiella indica* *Frankliniella occidentalis*, *Phytonemus pallidus* *Polyphagotarsonemus latus* *Tetranychus urticae* *Tetranychus urticae*	ABC ABC ABC CBC-ABC ABC	Kondo et al. (2020)
Cuba	*Amblyseius largoensis* *Neoseiulus longispinosus* *Phytoseiulus macropilis*	*Polyphagotarsonemus latus* *Tetranychus tumidus* *Panonychus citri*	ABC ABC ABC	Márquez et al. (2020) Van Lenteren and Bueno (2003)
Ecuador	*Neoseiulus californicus* *Phytoseiulus persimilis*	*Tetranychus urticae* *Tetranychus urticae*	ABC ABC	Suquilanda Valdivieso (2017)
Honduras	*Neoseiulus longispinosus* *Neoseiulus cucumeris* *Neoseiulus swirskii*	*Tetranychus* spp. Thrips Thrips and whiteflies	ABC ABC ABC	Trabanino et al. (2020)
Mexico	*Amblydromalus limonicus* *Amblyseius victoriensis* *Iphiseius degenerans* *Neoseiulus californicus* *Neoseiulus cucumeris* *Phytoseiulus persimilis* *Thyphlodromips swirskii* *Galendromus occidentalis*	*Frankliniella occidentalis* and *Thrips tabaci* *Phyllocoptruta oleivora* Thrips *Tetranychus urticae* *Frankliniella occidentalis* and *Bemisia tabaci* *Tetranychus urticae* *Trialeurodes vaporariorum*, *Bemisia tabaci*, *Frankliniella occidentalis*, *Thrips tabaci* Mites in citrus	CBC ABC ABC CBC CBC-ABC CBC-ABC CBC-ABC CBC-ABC CBC-ABC	Arredondo-Bernal and Rodríguez-Vélez (2020)
Peru	*Euseius victoriensis* *Euseius stipulatus* *Amblyseius largoensis* *Euseius scutalis*	*Panonychus citri* *Phyllocoptruta oleivora* *Polyphagotarsonemus latus* *Oligonychus punicae*	CBC-ABC CBC-ABC CBC-ABC CBC-ABC	Mujica and Whu 2020
Uruguay	*Amblyseius swirskii*	Thrips and whiteflies	CBC-ABC	Basso et al. 2020

^a^Entered as *Amblyseius chilensis* (Crouzel 1983) and reintroduced in 2007

## Experiences with *Neoseiulus californicus* in Argentina

*Neoseiulus californicus* is distributed in Europe, Japan, South Africa Canada, North America, and South America (Demite et al. 2022) and is used by augmentative releases in numerous countries, such as the Netherlands, Belgium, Spain, Israel, Japan, USA, Mexico, Brazil, Colombia, and Chile where it is massively raised and marketed.

Members of type II of the Phytoseiidae family have a preference for a broad range of tetranychid species, but they also feed and reproduce on mites of other groups, including Eriophyidae, Tarsonemidae, and Tydeoidea, and also on pollen (McMurtry et al. 2013; Khanamani et al. 2017; Soltaniyan et al. 2018, 2020). Commercial *Typha angustifolia* pollen Nutrimite® is an acceptable food for *N. californicus* because it enables this predator to develop and reach adulthood, as well as reproduce and have viable offspring, although their performance is significantly higher when it feeds on *T. urticae* (Pascua et al. 2020).

Strains from different geographic areas often differ in some population parameters and tolerance or susceptibility to certain factors, such as drought, insecticides, and winter conditions. In Argentina, there are no biofactories that produce this control agent, but several studies that have been carried out on its ecology suggest that it is possible to implement conservation biological control strategies, particularly in strawberry crops in the horticultural belt of La Plata, Buenos Aires (34°56′00″S, 57°57′00″W). In strawberry greenhouses, Greco et al. (1999) found both *T. urticae* and *N. californicus* populations were widely distributed, although, in general terms, *N. californicus* exhibited an uneven aggregation than *T. urticae*. This would create refugees for the prey, thus increasing the persistence of the system. The high spatial coincidence of *N. californicus* with *T. urticae* indicates an important dispersal capacity of the predator, and a high ability to detect leaflets with prey (Greco et al. 2005).

Several wild plants surrounding crops may provide temporary habitat and potential food sources for *N. californicus* in that region, in different seasons. Pollen from *Urtica urens* L., *Lamium amplexicaule* L., *Convolvulus arvensis* L., *Sonchus oleraceous* L., *Galega officinalis* L., allowed the development of *N. californicus* adult, but not a reproduction. Survival was 70–80% when fed on pollen from *S. oleraceus*, *G. officinalis*, and *C. arvensis*, 80–90% when fed on pollen from *U. urens* and *F. x ananassa*, and more than 90% when fed on *T. urticae* and pollen from *L. amplexicaule*. In autumn and winter, *U. urens*, *L. amplexicaule*, and *S. oleraceous* could promote the persistence of *N. californicus* when prey density in strawberry is low since this plant species provide supplementary food sources. In summer, pollen of *C. arvensis* and *G. officinalis* would contribute to the persistence of *N. californicus* when the strawberry crop is ending and offers scarce food resources. Although the pollen of these plants would not enable the predator population to increase, the presence of these plants in the vicinity of strawberries could contribute to the persistence of the *N. californicus* population and help to limit *T. urticae* growth when this pest begins to colonize the crop (Gugole Ottaviano et al. 2015).

The ability to resist winter conditions and periods of starvation is another characteristic of this strain. Greco et al. (2006) found a survival of 62.5% after 96 h of starvation, and it has recently been observed that females can survive 10 days without food (Alonso, personal communication). Although total fecundity decreased due to a reproductive diapause during starvation, the number of offspring produced by females after the periods of starvation was not significantly different from those fed females, they leveled at approximately 2.9 eggs per female per day. Besides, the fecundity of individuals of this *N. californicus* strain decreased significantly under winter conditions, but reproductive diapause might not be observed. In the laboratory, individuals exposed to winter conditions throughout the life cycle exhibited a long pre-oviposition period, and low oviposition rate but did not diapause. After being kept under winter conditions from larva to adult, when individuals were transferred to the optimal spring temperatures and lighting, the pre-oviposition period was shorter, and the fecundity was higher than under winter conditions. When it remained under spring conditions from larva to adult and was then transferred to the winter parameters during the first 15 days of adulthood, the pre-oviposition period was long and the oviposition rate was low. Once the optimal conditions were restored, the daily fecundity became similar to that of the individuals remaining under optimal conditions throughout the life cycle (Gugole Ottaviano et al. 2018).

All these features suggest that *N. californicus* is a good candidate for conservation biological control in La Plata, Buenos Aires. A management plan for *T. urticae* in strawberries, based on the natural control by *N. californicus*, and acaricide applications only when necessary was developed and validated (Greco et al. 2011). The plan has two components: a sampling protocol and a decision chart. Systematic presence-absence sampling of active *T. urticae* and *N. californicus* was used to predict prey and predator densities relying only on the proportion of *T. urticae*-infested leaflets, once the occurrence of the predator was detected in at least one of them (Greco et al. 2004). The decision chart was constructed taking into account the relative pest and predator densities and the pest’s rate of increase (Greco et al. 2005)*.* It determines the range in the proportion of *T. urticae*-infested leaflets that will require different actions: to use selective acaricides and re-check at 7 days, to take no action but re-check at 7 days, and to take no action but re-check at 14 days. The management plan is potentially effective and feasible, showing that natural populations of *N. californicus* can consistently produce strong top-down suppression of *T. urticae*. It was experimentally implemented in 11 commercial lots for 2 years, and it was found that spider mite densities remained low and similar to those of lots under conventional management. The management plan’s application reduced the usage of acaricides by 90% and needs much less time (25% less) than applying acaricide in an area of the same size (Greco et al. 2011). This biological conservation method, which reduces the frequency of acaricide usage to conserve the predator, might be supplemented by occasional augmentative releases, as shown by the same proposal to abolish chemical control for *T. urticae* in strawberries.

Other helpful knowledge for biological control through this agent in integrated management plans is the compatibility between this strategy and others, such as the use of resistant cultivars to *T. urticae*. Gugole Ottaviano et al. (2013) found that Festival and Albion could be suitable strawberry cultivars used in *T. urticae* management programs that include biological control by *N. californicus*. Ultimately, two other aspects are being investigated: (1) dispersal based on resource availability and (2) intraguild predation by *Orius insidiosus* Say (Hemiptera: Anthocoridae), the main predator of *Frankliniella occidentalis* (Pergande) (Thysanoptera: Thripidae); to determine the compatibility of these agents for the control of two crucial strawberry pests.

## Final remarks

Biological control using leaf-inhabiting phytoseiid has been booming during the last decades, since these predatory mites play a significant role in the biological control of mites and insect pests. Therefore, they have been the subject of intensive studies across the world, including several Latin American countries, mainly Brazil and México. These studies have resulted in an important number of new species described, as well as a wide knowledge of bioecology, geographic distribution, predation capacity, etc. Nevertheless, till now, few species are effectively used in biological control programs.

This fact highlights the need to continue the investigations on the ability of other, unknown species to be used in biological control through close collaboration between researchers and biocontrol companies. Still, different challenges such as developing better rearing systems to provide huge numbers of predators to farmers in different crop systems, training farmers to improve their understanding of the use of predators, and chemical control aiming to reinforce the use of conservation biological control.
