# Correction: Current Status of Phytoseiid Mites as Biological Control Agents in Latin America and Experiences from Argentina Using *Neoseiulus californicus*

**DOI:** 10.1007/s13744-024-01157-2

**Published:** 2024-04-10

**Authors:** Carlos Vásquez, Yelitza Coromoto Colmenárez, Nancy Greco, Mayra Ramos

**Affiliations:** 1 Faculty of Agronomical Sciences, Technical University of Ambato, Campus Querochaca, Province of Tungurahua Cevallos, Ecuador; 2 CABI Latin America, Botucatu, São Paulo Brazil; 3 Centro de Estudios Parasitológicos y de Vectores (CEPAVE-CCT-La Plata-CONICET-UNLP), La Plata, Argentina; 4 Tecnológico Nacional de México, Campus Los Reyes, Colonia Libertad, Los Reyes, Michoacán México

**Correction: Neotropical Entomology (2023) 52:240–250**

10.1007/s13744-023-01026-4

The article “Current Status of Phytoseiid Mites as Biological Control Agents in Latin America and Experiences from Argentina Using *Neoseiulus californicus“*, written by Carlos Vásquez, Yelitza Coromoto Colmenárez, Nancy Greco & Mayra Ramos, was originally published Online First without Open Access. After publication in volume 52, issue 2, page 240–250, the author decided to opt for Open Choice and to make the article an Open Access publication. Therefore, the copyright of the article has been changed to © The Author(s) 2023 and the article is forthwith distributed under the terms of the Creative Commons Attribution 4.0 International License, which permits use, sharing, adaptation, distribution and reproduction in any medium or format, as long as you give appropriate credit to the original author(s) and the source, provide a link to the Creative Commons licence, and indicate if changes were made.

The images or other third party material in this article are included in the article’s Creative Commons licence, unless indicated otherwise in a credit line to the material. If material is not included in the article’s Creative Commons licence and your intended use is not permitted by statutory regulation or exceeds the permitted use, you will need to obtain permission directly from the copyright holder.

To view a copy of this licence, visit http://creativecommons.org/licenses/by/4.0/

